# *In vitro* study: green synthesis and evaluation of MgO/C-dots/DOX phosphorescent nanocomposites for photodynamic/photocatalytic therapy of tumors

**DOI:** 10.3389/fbioe.2023.1286955

**Published:** 2023-11-22

**Authors:** M. Karimi, E. Sadeghi, M. Zahedifar, M. Nejati, H. Mirzaei, Michael R. Hamblin

**Affiliations:** ^1^ Institute of Nanoscience and Nanotechnology, University of Kashan, Kashan, Iran; ^2^ Department of Physics, University of Kashan, Kashan, Iran; ^3^ Anatomical Sciences Research Center, Kashan University of Medical Sciences, Kashan, Iran; ^4^ Research Center for Biochemistry and Nutrition in Metabolic Diseases, Institute for Basic Sciences, Kashan University of Medical Sciences, Kashan, Iran; ^5^ Department of Dermatology, Harvard Medical School, Boston, MA, United States; ^6^ Wellman Center for Photomedicine, Massachusetts General Hospital, Boston, MA, United States

**Keywords:** MgO nanoparticles, photodynamic therapy, *in vitro*, anti-cancer, phototoxicity

## Abstract

MgO nanoparticles (NPs) and carbon dots (C-dots) were synthesized by co-precipitation and hydrothermal techniques. In the next step, as-synthesized NPs were modified by C-dots. Then, polyethylene glycol (PEG) was conjugated with MgO/Cdots. Finally, Doxorubicin (Dox) as an anticancer drug was loaded on MgO/Cdots/PEG nanocomposites. The XRD pattern showed the characteristic peaks of C-dots and MgO. The FTIR spectrum showed that MgO/C-dots possessed the carboxyl functional groups, allowing DOX to be loaded onto MgO/C-dots/PEG through hydrogen bonds. The particle size of MgO, C-dots, MgO/C-dots, and MgO/C-dots/PEG/DOX was 20–30, 5–10, 30–40, and 100–130 nm, respectively, using TEM, DLS, and FESEM techniques. MgO, MgO/C-dots, and MgO/C-dots/DOX were fluorescent NPs when excited by a UV source. Anthracene and methylene blue were used as fluorescent probes to identify the reactive oxygen species (ROS) produced by UV excitation. The activity of MgO/C-dots and MgO/C-dots/DOX against colorectal cancer (C26) cells, after repeated 5-min illumination with both UV-light and red light LEDs, were measured by MTT assay. C26 cancer cells incubated with DOX-loaded MgO/C-dots and exposed to either wavelength (UV and red) killed ∼70% of cells. The green synthesized nanocomposites could act as anti-cancer photosensitizers probably by a photocatalytic mechanism.

## 1 Introduction

Photodynamic therapy (PDT) has attracted considerable attention in recent years owing to its non-invasive nature, avoidance of damage to healthy parts of the body, and its potential for extremely precise tumor therapy (Han et al., 2015; Lucky et al., 2015; Seidl et al., 2016). The drawbacks of routine chemotherapy like severe side effects or onset of multidrug resistance in some malignant tumors may therefore be avoided. PDT has negligible systemic toxicity in tissues that are not exposed to light. However, there is significant cytotoxicity in light-exposed tumors due to the photochemical production of reactive oxygen species (ROS) (Castano et al., 2004; Wan et al., 2014; He et al., 2015; Mo et al., 2022). The photosensitizer (PS) molecule absorbs light at a certain wavelength and is excited to the first excited state (S_1_). From there, it must undergo intersystem crossing (ISC) to form the triplet excited state (T_1_) or return to the ground state (S) through fluorescence or heat generation. The long-lived T_1_ PS can react with oxygen via two mechanisms (type I and II), generating a variety of ROS (Weston et al., 2016; Castilho et al., 2017; Karimi et al., 2021; Wang et al., 2022; Khorsandi et al., 2022; Liang et al., 2022). PDT can be used as therapeutic option for a variety of cancers, including esophageal, malignant glioma, bladder cancer, pharyngeal and oral cancer, lung cancer, skin cancer, liver, breast, and pancreatic cancer (Lu et al., 2019). Sufficient oxygen must be present inside the tumor, but compared to normal tissues, deep tumors frequently exhibit pronounced hypoxia. Additionally, hypoxia will be made worse since the rate of oxygen consumption in PDT is higher than oxygen diffusion into the irradiated region. Therefore, tissue hypoxia will impair ROS generation during PDT and lessen the therapeutic effects.

In an ideal situation, there would be enough oxygen in the tumor tissues for PDT to effectively create a large amount of ROS and cause significant cytotoxicity (Kalka et al., 2000; Stockwell et al., 2017). Some recent advances in innovative deep tissue PDT have been based on various nanoparticles (NPs) as described in these reviews (Hu et al., 2015; Tavakkoli et al., 2018). In general, two types of PSs have been described for PDT. One type is small molecule PSs (based on porphyrins or other tetrapyrrole compounds), while the other is based on inorganic NPs, such as binary oxide semiconductor materials (e.g., ZnO, TiO_2_) (Li et al., 2018; Wang et al., 2022; Sharma et al., 2022; Zhang et al., 2022). These NPs mainly carry out photocatalysis based on electron transfer reactions to oxygen to initially produce superoxide followed by hydroxyl radicals.

Doxorubicin (DOX) is a well-known anticancer agent employed in the management of melanoma, lymphoma, sarcomas, and carcinomas (Chang et al., 2014). DOX and PSs can be used together either as a co-treatment or bound to a nanocarrier platform which increases the efficiency of DOX (Grebinyk et al., 2019).

MgO is a common non-toxic metal oxide, employed as a catalyst, modifier, an additive for superconducting and refractory materials, as well as a stabilization agent in the pharmaceutical industry (Srisuvetha et al., 2020; Yathisha et al., 2020; Razmara et al., 2021). Once MgO is prepared as nanoscale NPs, its beneficial properties, such as its negligible electro-conductivity, good catalytic behavior, and significant thermal stability, may be used in the biological applications. Many techniques have been used to create the MgO NPs, including sol-gel, aqueous wet chemical, flame spray pyrolysis, laser vaporization, hydrothermal, chemical gas phase deposition, surfactant methods, and combustion aerosol methods (Silva et al., 2022). However, these methods may be expensive, produce toxic byproducts, or even produce very dangerous compounds. Green synthesis techniques have since emerged as a good substitute for traditional methodology (Schwab et al., 2021). The employment of green synthesis methods to fabricate NPs has many advantages, such as cost-effectiveness, environmental friendliness, and end-product purity. Green synthesized NPs may then be applied in nanomedical, nanopharmaceutical, nanooptoelectronic, and semiconductor industries (Patwardhan et al., 2018; Zikalala et al., 2018).

Carbon dots (C-dots) are unique nanomaterials, which have generated considerable interest in the biological applications. Considerable research has been conducted on C-dot-based nanocomposites for cancer treatment, particularly PDT since they can act not only as PS themselves but also as nanoplatforms to deliver the PSs (Fan et al., 2019; Xu et al., 2021). Much work has been done on C-dot-based nanomaterials for PDT, and PDT combination treatment for cancer. Graphite quantum dots (GQDs) have been used for drug delivery and PDT (Markovic et al., 2012; Ge et al., 2014; Roeinfard et al., 2021). Some composite materials include GQD@MnO_2_ (Meng et al., 2018), Ag-GQDs/DOX (Habiba et al., 2016), and N-GQD-DOX-APTES (Ju et al., 2019). Additionally, Wang et al. (2022) demonstrated the effective nuclear transport of DOX via DOX-GQD conjugates. According to this study, the conjugates might significantly boost the ability of DOX for DNA cleavage. DOX-GQD conjugates significantly increased the cytotoxicity of DOX, and the nuclear uptake of DOX in drug-resistant cancer cells, demonstrating the ability of C-dot conjugates to boost the chemotherapeutic effectiveness of anticancer drugs (Bansal et al., 2019a; Bansal et al., 2019b).

**SCHEME 1:**
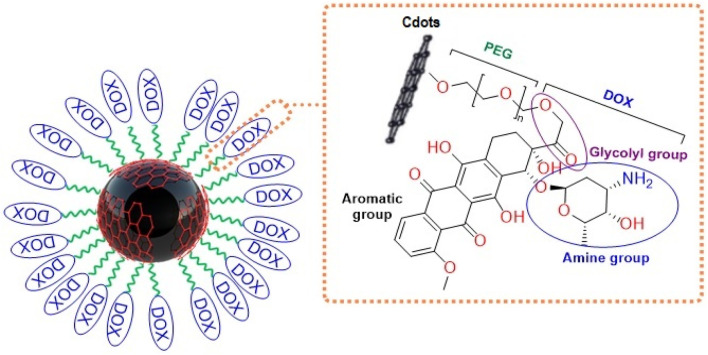
Preparation of DOX-Loaded MgO/Cdots.

In the present study, first MgO NPs were green synthesized, their surface was decorated by green synthesized C-dots and finally functionalized with polyethylene glycol (PEG) (Scheme 1). Magnesium oxide fluorescent nanocomposites (MgO/C-dots) were loaded with DOX and tested for biomedical purposes. We compared MgO, MgO/C-dots, and MgO/C-dots/DOX NPs as photosensitizers for PDT. We used a C26 *in vitro* cancer cell model to look at the dark toxicity of the materials, the acute phototoxicity of MgO, MgO/C-dots, and MgO/C-dots/DOX exposed to either blue-light or red-light LED illumination.

## 2 Materials and methods

### 2.1 Production of *Artemisia absinthium* L leaf extract

Plant extract-mediated production of NPs uses extracts from different parts of the leaf, rhizome, root, stem, bark, flower peel, and fruit, with no need for extrinsic capping substances, surfactants, or templates. The plant-derived extract includes phytochemicals and biomolecules, such as flavonoids, polyphenols, phenolic acids, terpenoids, and alcohols, which operate as stabilizing and reducing agents for metal ions or their precursors. Such biomolecules are classified into two types: 1) redox agents for the reduction of metals and 2) capping substances that aid in both the non-agglomeration and surface modification of NPs (Verma et al., 2021).

*A*. *absinthium* L fresh leaves were procured and thrice rinsed with distilled water (DW). The fresh leaves (5 g) were broken up into small pieces, combined with 50 mL of DW, mixed, and heated at 80°C–95°C for 20 min. The Whatman filter paper was used to filter the resultant solution before it was used to create MgO NPs.

### 2.2 Preparation of MgO/C-dots nanocomposites

The green synthesis of MgO NPs was done by a co-precipitation method. Mg(NO_3_)_2_.6H_2_O (Merck high purity, Germany) and *A. absinthium* L extract were used to synthesize MgO NPs.

**SCHEME 2:**
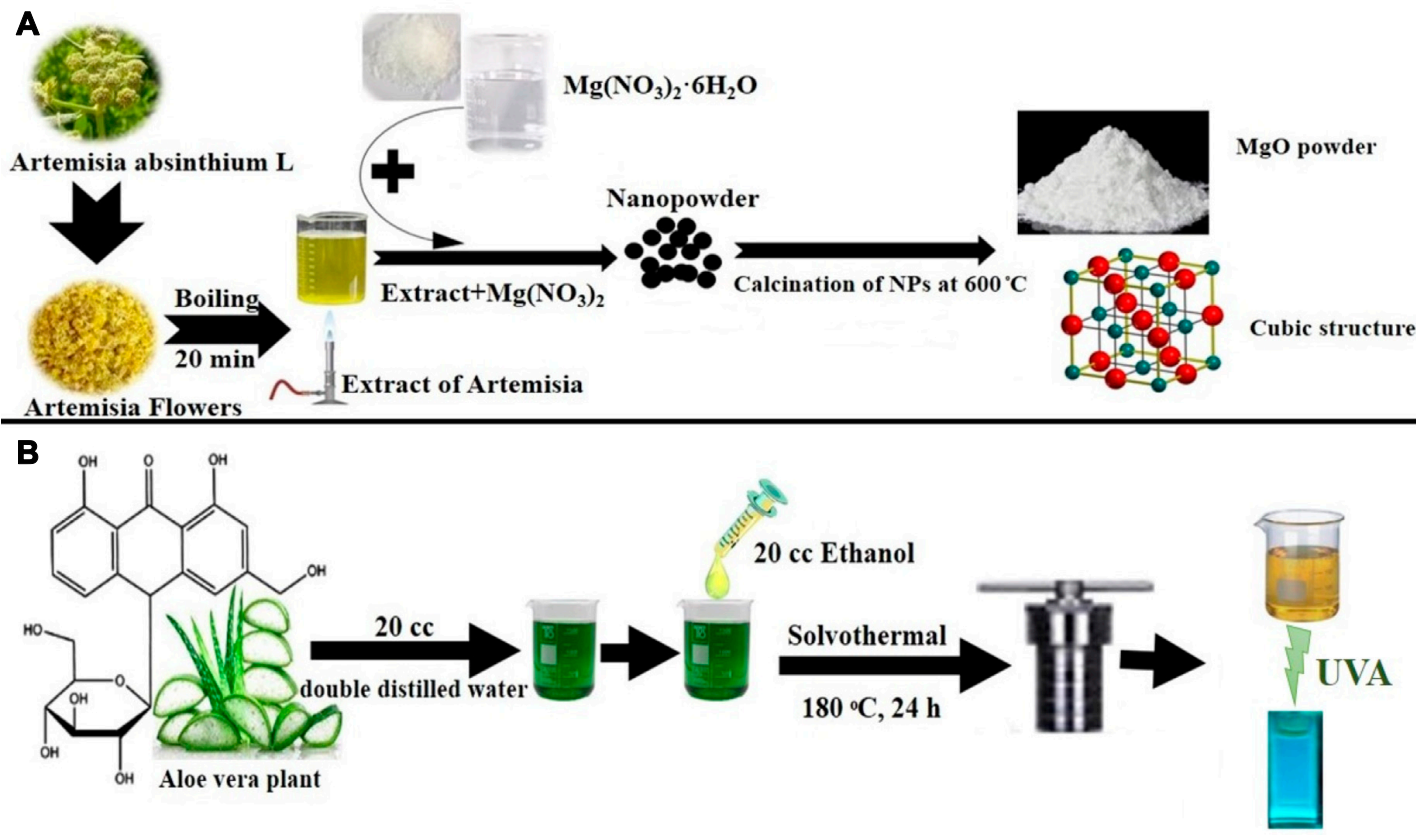
**(A)** Green synthesis of MgO NPs using a coprecipitation method. **(B)** Green synthesis of C-dots using a solvothermal method.

For the synthesis, Mg(NO_3_)_2_.6H_2_O (1.28 g) was dispersed in double distilled water (10 mL) for an hour at room temperature. Then the temperature was increased to 40°C, and *A. absinthium* L extract was added dropwise to the suspension, followed by stirring for 1 h. The mixture was centrifuged and washed three times with distilled water (3,500 rpm). The MgO NPs were dried at 80°C and then calcined at 600°C to obtain a powder. Scheme 2A. Depicts the production of MgO NPs using the coprecipitation method.

The C-dots were produced using a straightforward, practical one-step hydrothermal green process from *Aloe vera* skin. Double-distilled water was used to clean the Aloe vera skin, and it was then dried at 40°C. Then, 1.0 g of skin and 20 mL double-distilled water plus 20 mL ethanol were placed in a stainless steel Teflon-covered autoclave and heated for 24 h at 180°C. The product was filtered, followed by centrifuging at 12,000 rpm for 5 min after cooling to ambient temperature as shown in Scheme 2B.

### 2.3 Synthesis of MgO/Cdots

In order to prepare MgO/C-dots nanocomposites, 0.5 g of MgO NPs was added to 5 mL of C-dots suspension and agitated for 60 min. The resultant suspension was heated at 170°C for 3 h inside an autoclave. Finally, the product was harvested by filtering and dried at 50°C.

### 2.4 Synthesis of MgO/Cdots/PEG 6000

As-synthesized NPs should be adjusted to avoid aggregation and sedimentation. Covering NPs with polymeric materials is a well-known technique for modifying them. In this particular research project, the modifying agent that was used was PEG 6000. 10 mL of MgO/C-dots solution (0.1 g MgO/C-dots) was used to conjugate with 0.01 g PEG 6000 to NPs.

### 2.5 Loading MgO-C-dots with DOX

The MgO@C-dots–PEG compound could be used to transport drugs. To load DOX onto the carrier, a Double ionized water solution of DOX was added to a suspension of MgO@C-dots–PEG. The suspension was stirred for 24 h at room temperature in the dark.

### 2.6 Characterization

#### 2.6.1 X-ray diffraction (XRD)

To investigate the structure of the crystal, an X-ray diffraction analysis was carried out (model: Philips X’pert Pro MPP with Cu K radiation filtered by Ni, and = 0.1540 nm). The Scherrer formula, which is given in the following way, was used to determine the average crystallite size (D) of Cdots, MgO, MgO/Cdots, and MgO/Cdots/DOX NPs. In this particular format, the formula was applied as follows:

$$D=\frac{K\lambda }{\beta \mathrm{COS}\theta }$$ (1a)

Where k is the form factor (k = 0.9), *β* is the full width half maximum (fwhm), and θ is the angle at which the light is coming in.

#### 2.6.2 Scanning electron microscopy (SEM), and energy-dispersive X-ray spectroscopy (EDS)

Scanning electron microscope (TESCAN Mira 3-XMU; SEM; model) was used to analyze the NPs morphology, content, and particle size. An energy-dispersive spectrometer (EDS)-equipped.

#### 2.6.3 Transmission electron microscopy (TEM), dynamic light scattering (DLS)

Transmission electron microscope (TEM; model: Zeiss EM900) and DLS (VASCO/CORDOVAN TECHNOLOGIES/FRANCE). The DLS results corroborated the size distribution of synthesized NPs.

#### 2.6.4 FTIR spectroscopy

The existence of functional groups was investigated using a Fourier transform infrared (FT-IR; Magna-IR550) spectrometer.

#### 2.6.5 Absorbance, fluorescence, fluorescence energy transfer (FRET), phosphorescence

In order to look into the optical characteristics of a material at ambient temperature, an ultraviolet-visible (UV-Vis; UVS-2500, PHYSTEC/Iran) spectrometer was employed to measure the spectrum from 200 to800 nm. Spectra of photoluminescence (PL) and fluorescence energy transfer (FRET) were captured at room temperature using a Perkin-Elmer LS55 spectrometer equipped with a photomultiplier tube and Xenon lamp. Afterglow measurements were done with a commercial TLD reader, Harshaw Model 4,500.

### 2.7 Photochemical mechanisms

To estimate singlet oxygen production, anthracene was utilized as a quenching probe. Anthracene (0.002 g) was dissolved in ethanol. The absorption spectra of anthracene were recorded using a UV-vis spectrometer after anthracene solution (0.06 mL) was added to free photosensitizer and NP compounds (Ps-NPs) prior to irradiation. The solutions were exposed to UVA light for 30 min. The absorption spectra were re-determined, and the amount of singlet oxygen produced was calculated by measuring the reduced absorbance. To detect free radicals, methylene blue (MB) was used as a hydroxyl radical-specific monitoring probe. Initially, 10 mL DI-water was mixed with 0.002 g of MB and 1.5 mL of MB solution was added to free photosensitizer and NPs (at neutral pH) before irradiating the samples with UVA for 30 min. Then the MB absorption was recorded using a UV-vis spectrometer.

### 2.8 Cell culture

The Cell Bank of Pasteur Institute of Tehran, Iran, provided a C26 mouse colon cancer cell line. Cells were maintained in RPMI-1640 medium (Gibco; Thermo Fisher Scientific, Inc., Waltham, MA, United States) supplemented with 10% fetal bovine serum (Gibco; Thermo Fisher Scientific, Inc., Waltham, MA, United States) and cultured in a humidified incubator containing 5% CO_2_ at 37°C.

### 2.9 *In vitro* light-mediated cell killing

The following light sources were employed. UV light (manufacturer, wavelength (364 nm), power density (0.21 mW/cm^2^), illumination time (10 min), and energy density (30 mJ/cm^2^). Red light (manufacturer, wavelength (600–630 nm), power density (.08 mW/cm^2^), illumination time (10 min), energy density (48 mJ/cm^2^) The cells (1 × 10^4^) were plated in media (200 μL) in a 96-well plate and incubated for 24 h. After reaching sufficient density, C26 cells were exposed to varying concentrations (expressed as µM of MgO or DOX) of free doxorubicin (DOX), MgO@C-dots, and MgO@C-dots-DOX. After incubation for 24 h, the cells were exposed to dark, UV light (30 mJ/cm^2^), or red light (48 mJ/cm^2^). Cells were returned to the incubator for an additional 24 h when viability was determined by the MTT assay. MTT solution (20 μL, 5 mg/mL) was added to all wells and incubated for 3 h. The media containing MTT was replaced by DMSO (100 μL) and re-incubated for 30 min. The optical density was read at 570 nm. The cytotoxicity was reported as 50% inhibitory concentration (IC50).

### 2.10 Statistical analysis

Data is demonstrated in the form of mean ± SD. SPSS 13.0 as well as GraphPad Prism 8.0 were used to analyze data. For comparing two groups, we used paired-samples tukey test; for comparing more than two groups, ANOVA was used. The statistical significance was set at *p < .*05.

## 3 Results and discussion

### 3.1 X-ray diffraction (XRD) and energy-dispersive X-ray spectroscopy (EDS)

**FIGURE 1:**
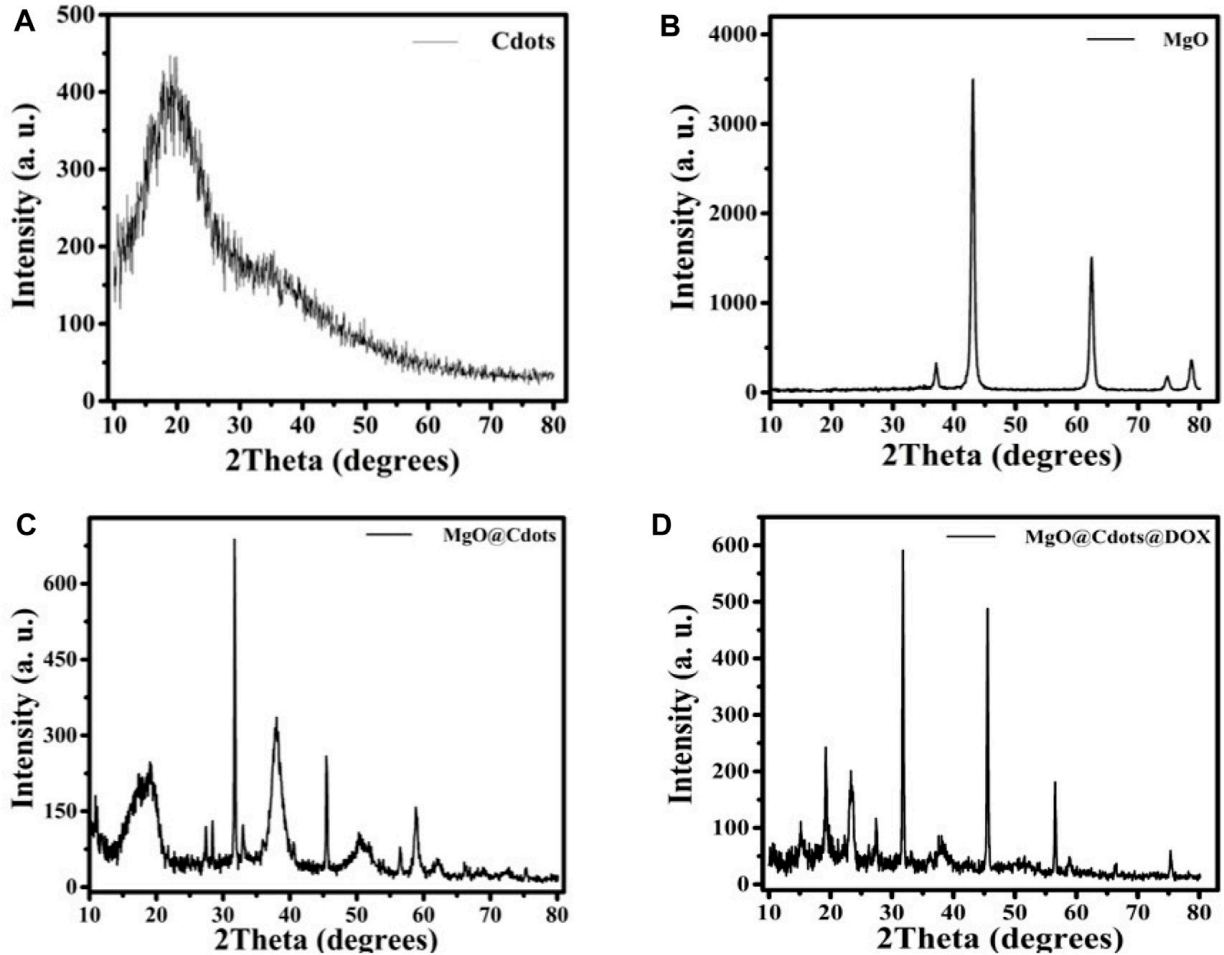
XRD patterns of **(A)** C-dots, **(B)** MgO, **(C)** MgO/C-dots and **(D)** MgO/C-dots/DOX.

Figure 1 displays the C-dot XRD patterns. The 24° (2θ) XRD single broad (002) peak showed the presence of an extremely small carbogenic core in C-dots (Liang et al., 2013; Pandiyan et al., 2020). XRD patterns of MgO NPs calcined at 600°C. showed peaks at 2θ° = 36.99°, 43.05°, 62.49°, 74.91°, and 78.81° assigned to MgO (111), (200), (210), (220), (311), and (222) planes (JCPDS card no. 01-074-1225) (Panchal et al., 2022; Kumari et al., 2023). The XRD patterns captured from MgO/C-dots NPs showed that coating the MgO surface with C-dots had no effect on the crystalline phase of the mineral. Further, the C-dots pattern at 2θ = 24° in the MgO/C-dots nanocomposite, confirmed MgO surface decoration with C-dots. In addition, a shift occurred in the peaks towards higher 2θ° = 37.29°, 45.25°, 64.89°, 75.81°, and 79.89° for the MgO/C-dots sample, corresponding to the lattice strain because of C-dots decoration on the MgO surface. A weak pattern appeared for C-dots related to the trace level of C-dots in MgO (111), (200), (210), (220), (311), and (222) planes (JCPDS card no. 01-074-1225) (gamal El-Sham and y, 2020). On the C-dots surface. Using the Scherer equation, the crystalline size obtained for C-dots was approximately 6 nm, and for MgO was approximately 20 nm. The relevant lattice factor of the (200) plane was estimated to be a = 4.21 Å. Furthermore, the mean crystalline size was about 20 nm. The crystalline size obtained for the MgO/C-dots was about 25 nm. The crystalline size obtained for the MgO/C-dots/DOX was about 110 nm, and no additional phase appeared on the XRD pattern. This corresponded to the accumulation of very fine carbon particles in NPs and drugs.

**FIGURE 2:**
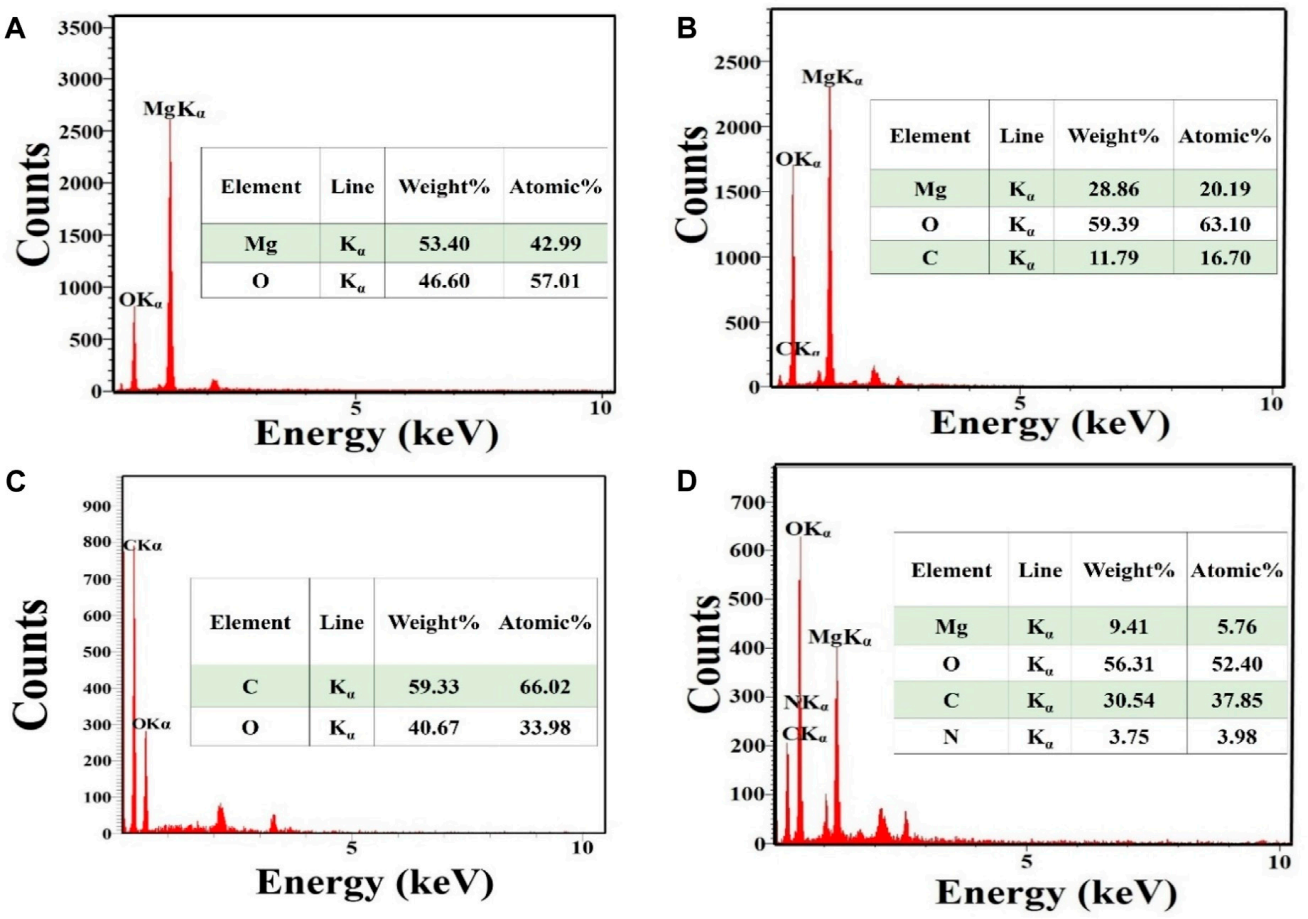
EDS spectra and elemental analysis. **(A)** MgO, **(B)** MgO/C-dots **(C)** C-dots, **(D)** MgO/C-dots/DOX.

**FIGURE 3:**
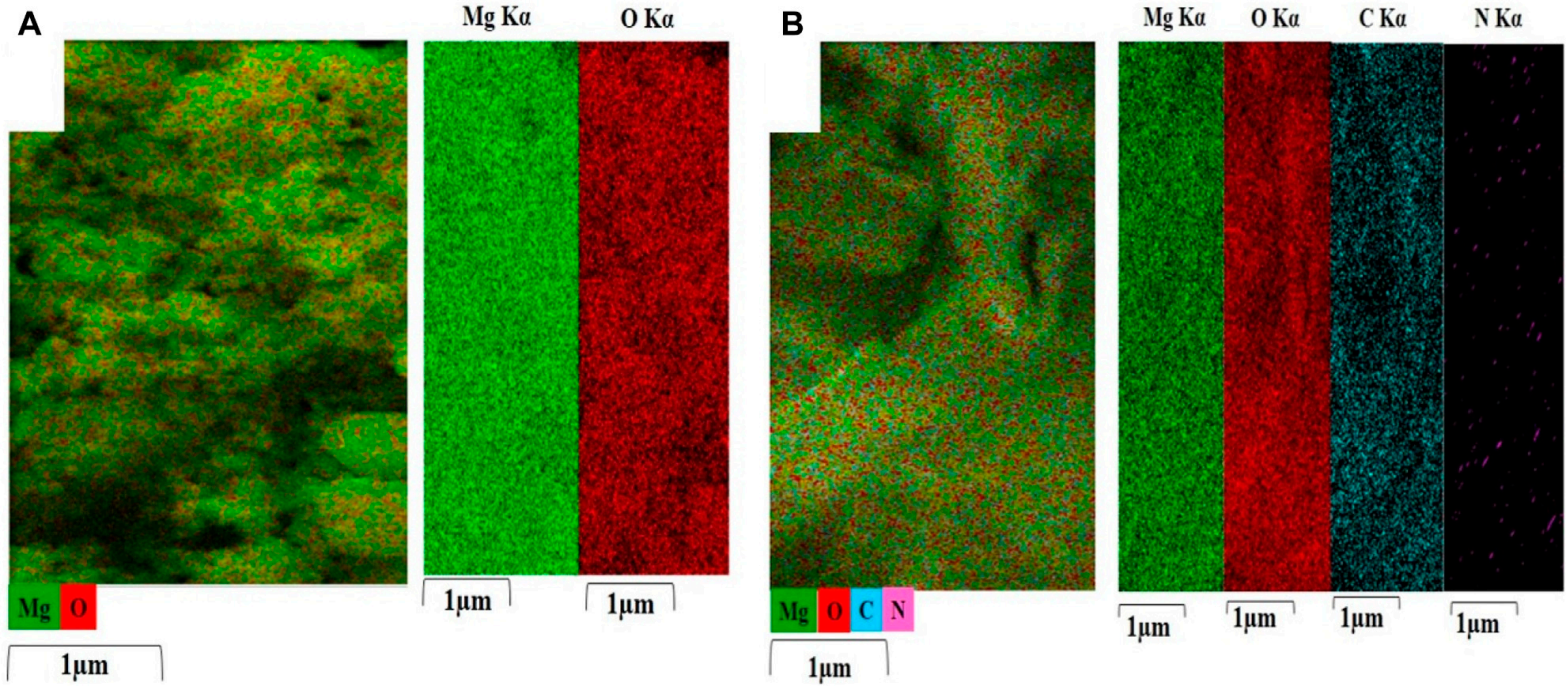
EDS elemental maps (Mg, O, C, and N) were obtained from **(A)** MgO, and **(B)** MgO/C-dots/DOX. Each map is displayed on a relative intensity scale over 1 μm where the color of each element is bright (element-rich) or dark (element-deficient).

Figure 2A shows the EDS spectrum captured from MgO. This showed the presence of Mg and O elements and there were no other impurities in the material. The proportion of Mg and O elements was 42.99% and 57.01% respectively. Figure 2B displays the EDS spectrum captured from MgO/C-dots NPs. This showed the presence of Mg, O, and C elements with relevant proportions of 20.19%, 63.10%, and 16.70% respectively. Figure 2C displays the EDS spectrum captured from the C-dots. This confirmed C and O elements with proportions of 66.02%, and 33.98% respectively. Figure 2D shows the EDS spectrum of the MgO/C-dots/DOX NPs. The presence of the elements Mg, O, C, N, and C with proportions of 5.76%, 52.40%, 37.85%, and 3.98% respectively. There were no other impurities in the material. Moreover, the mapping-EDS results (Figures 3A, B) showed the elements in the MgO and MgO/C-dots/DOX nanocomposite. According to the results, all elements (Mg, O, C, and N) were well distributed throughout the MgO/C-dots/DOX nanocomposite.

### 3.2 SEM, TEM and DLS analysis

**FIGURE 4:**
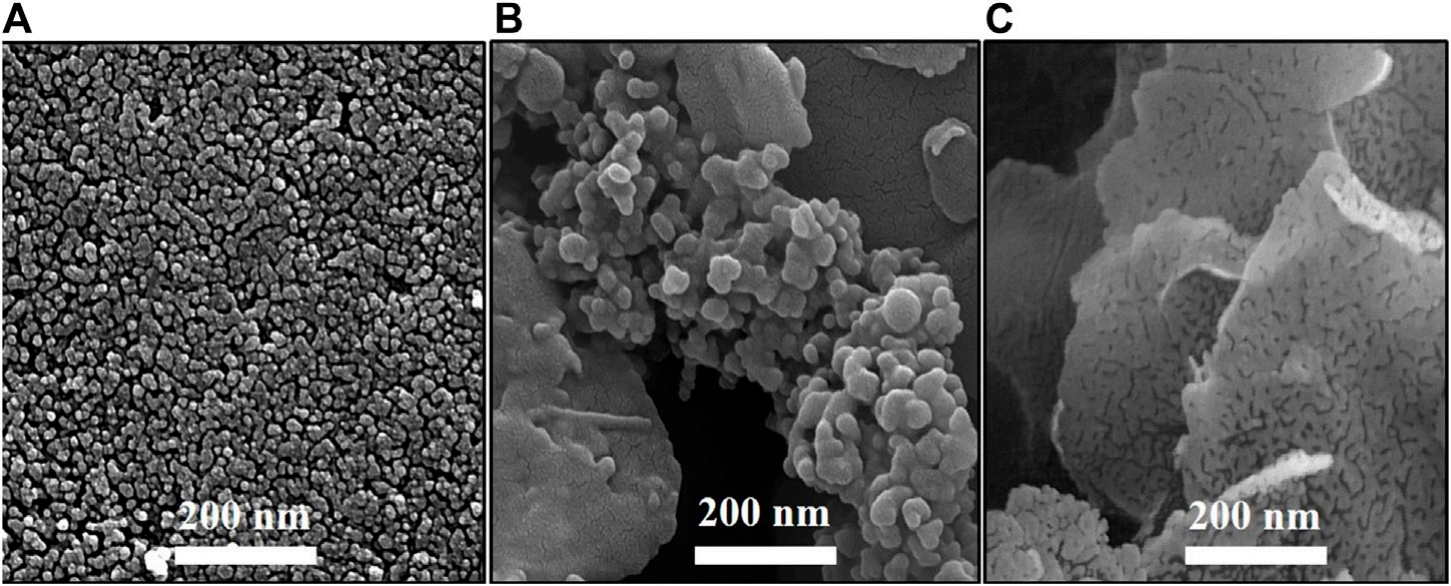
FESEM images of **(A)** MgO, **(B)** MgO/C-dots and **(C)** MgO/C-dots/DOX.

The particle shape and size are the main parameters affecting the biological properties. Hence, determining the shape and size of the MgO, MgO/C-dots, and MgO/C-dots/DOX NPs is important for anticancer applications. FESEM images were employed to explore the size and shape of MgO, MgO/C-dots, and MgO/C-dots/DOX NPs. Figure 4 illustrates the FESEM images captured for specimens a-c. In these samples, the capping agent was *A. absinthium* L. A spherical morphology was found for the MgO NPs. Homogeneity is also an important parameter for anticancer applications. The homogenous spherical shape can be seen in Figure 4A confirming the product homogeneity, and the sphere diameter was estimated to be 30 nm for MgO NPs. C-dots were used to decorate the MgO NPs in order to increase the efficiency of ROS generation, and this increased the size to about 37 nm. In Figure 4B some agglomeration can be seen in the MgO NPs, possibly due to the presence of C-dots. Very small particles possess greater surface energy and a further tendency towards agglomeration. In Figure 4C the surface of the MgO@C-dots was modified using PEG and then loaded with the drug doxorubicin which led to the NP size becoming approximately 125 nm. The MgO, C-dots, MgO/C-dots, and MgO/C-dots/DOX NPs were monodisperse with a mean hydrodynamic diameter of 37 nm, 8 nm, 40 nm, and 125 nm, respectively, which is considered appropriate for pharmaceutical applications (Caputo et al., 2019).

**FIGURE 5:**
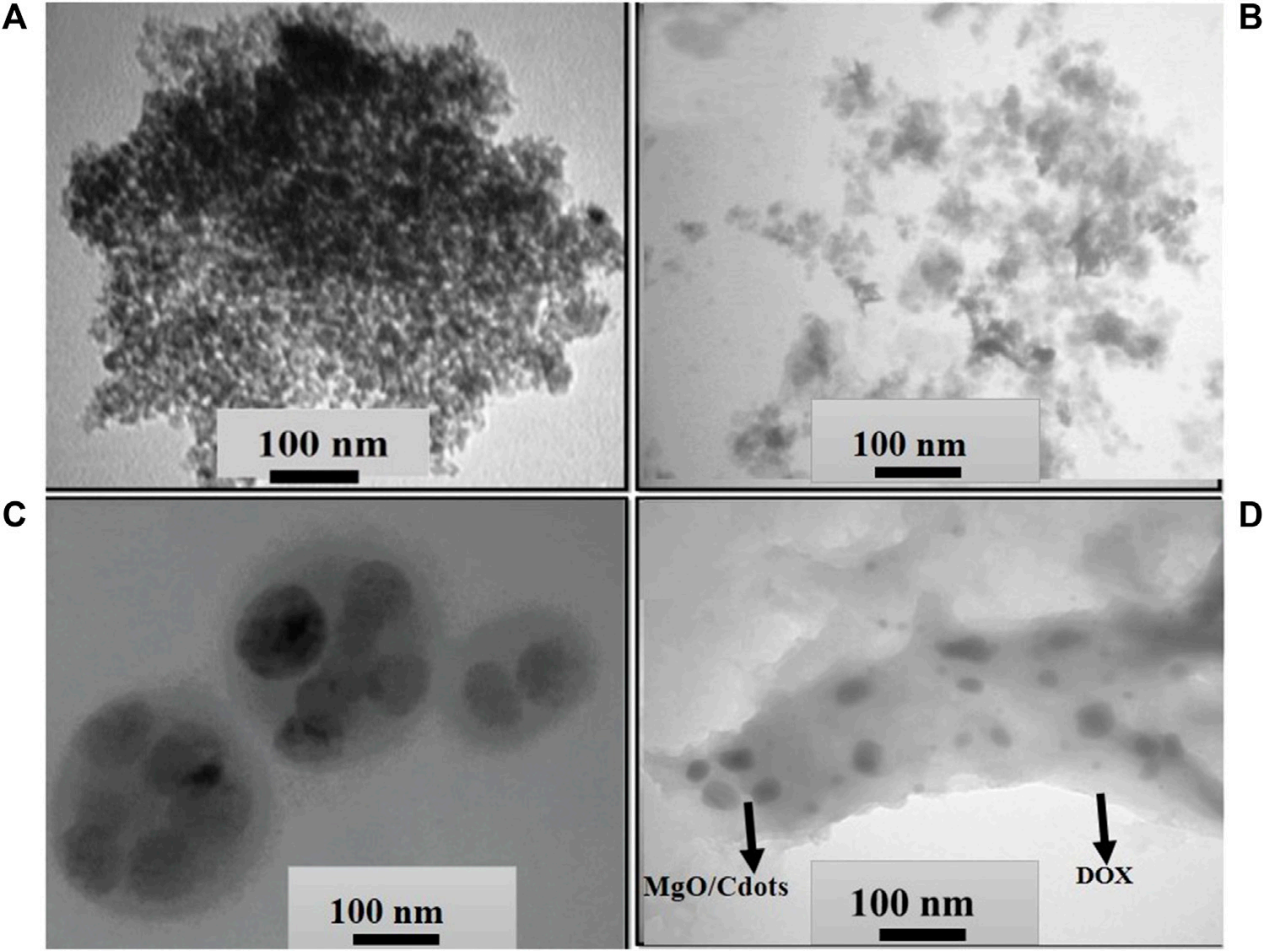
TEM images of **(A)** C-dots, **(B)** MgO NPs, **(C)** MgO/C-dots and **(D)** MgO/C-dots/DOX.

To explore the shape and size of NPs, TEM micrographs were employed. The TEM micrograph of C-dots is shown in Figure 5A. The C-dots showed a uniform spherical shape and a homogeneous structure (Figure 5A). These C-dots NPs had a particle size that varied from 3 to 10 nm, with a mean value of ∼7 nm. This value is consistent with the particle size reported in references (Pandiyan et al., 2020; Roeinfard et al., 2021). TEM images of MgO/C-dots and MgO/C-dots/DOX NPs are shown in Figures 5B, C. The MgO NPs are shown in Figure 5B, with a spherical shape and a uniform arrangement and distribution. The particle size was around 35 nm with no agglomeration, indicating that the co-precipitation process was successful in producing MgO NPs free of agglomeration. However, it can be seen that when C-dots were attached to the surface of MgO, the particle size increased to 40 nm as seen in Figure 5C. When MgO NPs were decorated with C-dots, the surface was modified with PEG and loaded with DOX, as shown in Figure 5D, the size of the NPs was 130 nm. Both FESEM and DLS measurements supported these results.

**FIGURE 6:**
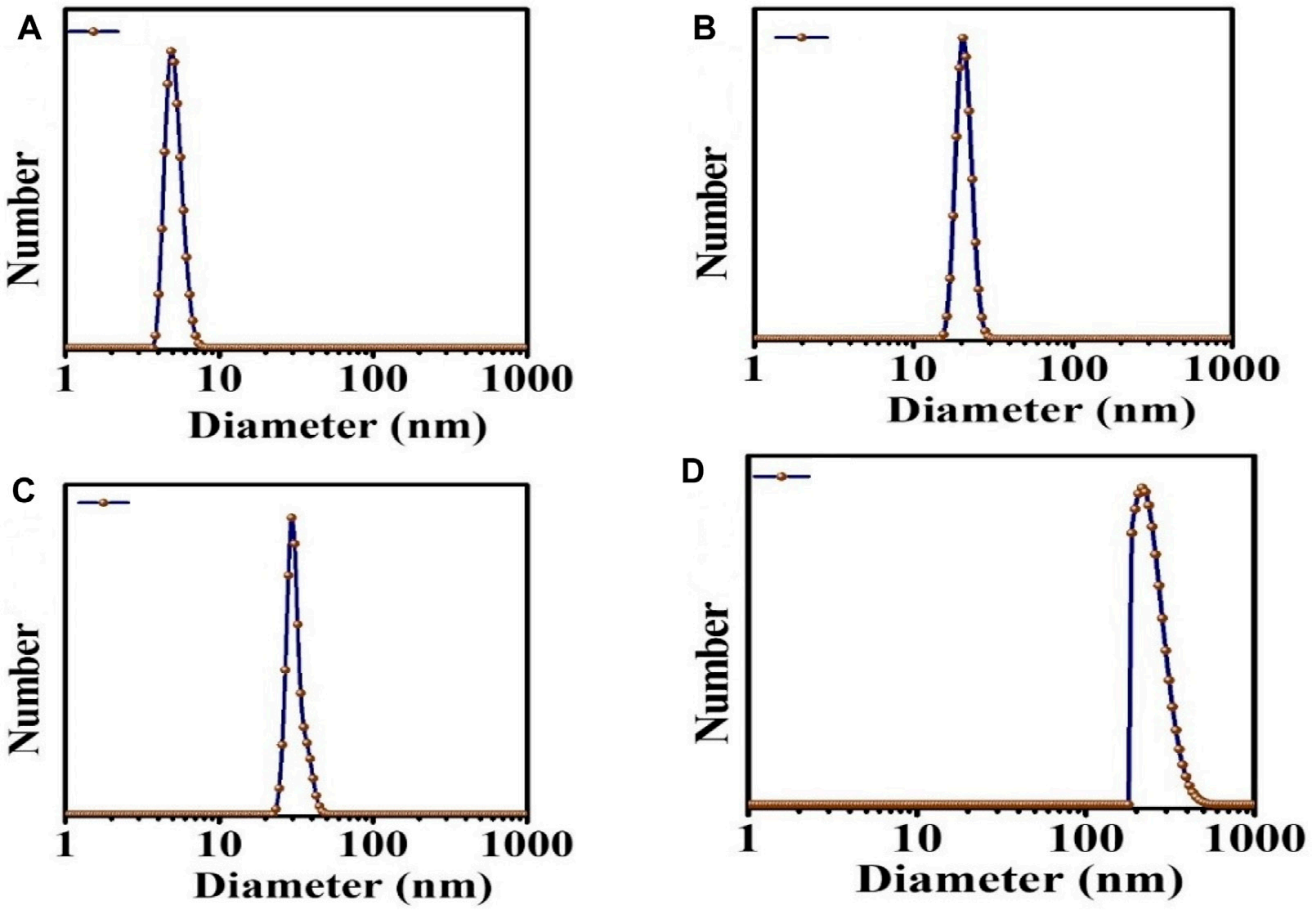
DLS analysis **(A)** C-dots, **(B)** MgO, **(C)** MgO/C-dots, and **(D)** MgO/C-dots/DOX.

DLS was carried out on samples inside a cuvette. This test was done to confirm the size distribution of each type of NPs. There was only one sharp peak, which means that only one type of particle is present in our samples. In Figures 6A–D, it can be seen the mean size distribution of C-dots NPs was 3–10 nm, MgO NPs were 40 nm, MgO/C-dots NPs were 45 nm, and MgO/C-dots/DOX NPs was 130 nm.

The sizes determined by TEM were larger than those determined by SEM analysis (Figure 4). This is because SEM is conducted on materials in the solid state, but DLS measures the hydrodynamic diameter of the NPs in suspension. The hydrodynamic diameter provides information on the core particle size, which is modified by coated material on the surface as well as the absorbed solvent layer, which vibrates due to Brownian motion. DLS analysis yielded a PDI of 0.5, which indicates a homogeneous size distribution of the particles.

### 3.3 FTIR study of MgO NPs and MgO modified by C-dots, PEG, and DOX

**FIGURE 7:**
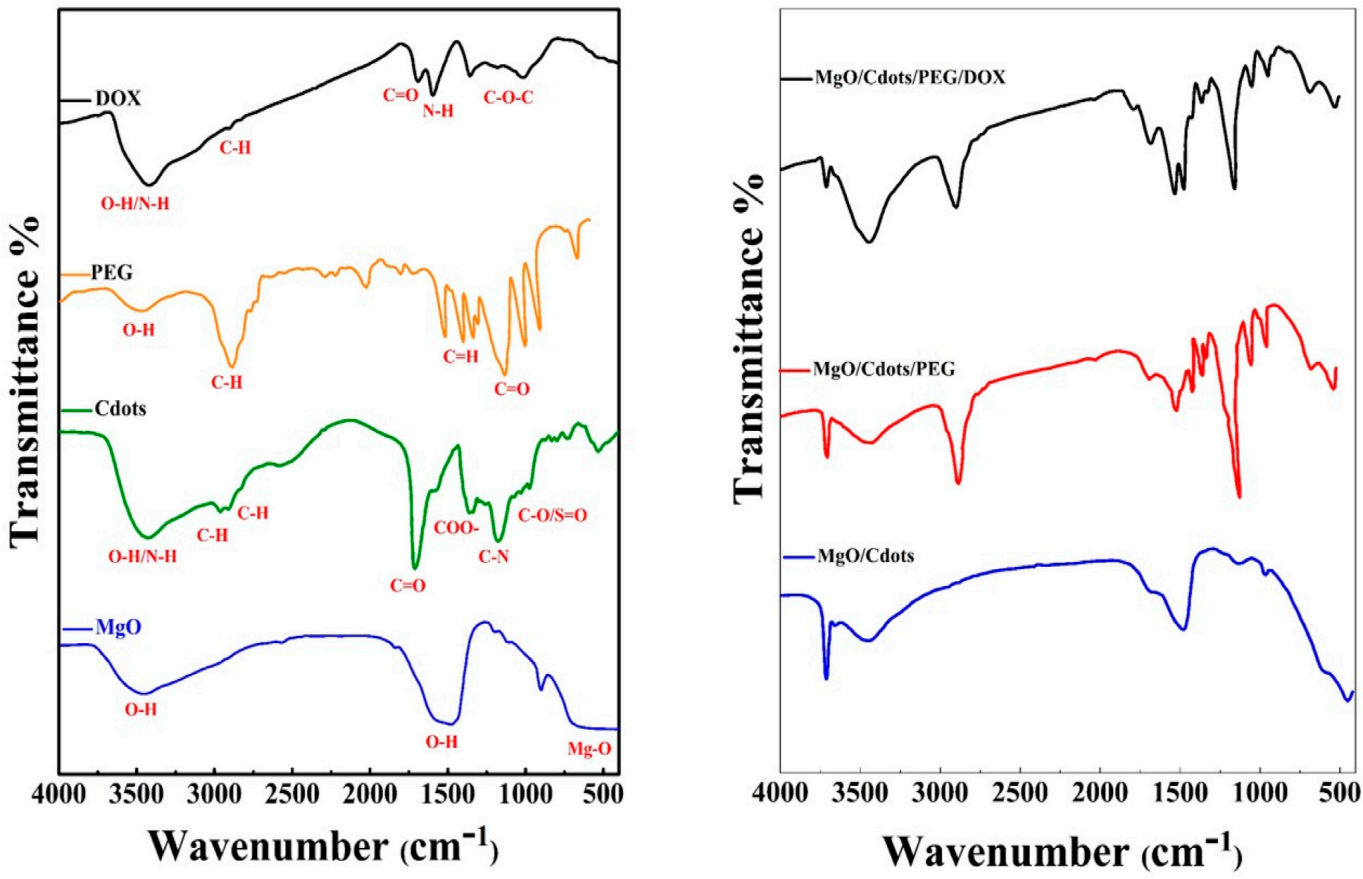
FTIR spectra of **(A)** MgO NPs, C-dots, PEG (pure), and DOX. **(B)** MgO–C-dots, MgO NPs–C-dots–PEG, MgO–C-dots–PEG–DOX.

FTIR was used to characterize MgO NPs, C-dots, PEG, DOX, MgO/C-dots, MgO/C-dots/PEG, and MgO/C-dots/PEG/DOX by identifying functional groups. Figure 7 shows all the obtained spectra. For pure MgO, its surface acid-base properties mean that MgO chemisorbs H_2_O and CO_2_ molecules from the atmosphere. Therefore, the modification of MgO–NPs by attachment of C-dots could optimize the photocatalytic activity. The Mg–O absorption band of MgO NPs can be seen at 400–700 cm^−1^. The stretching vibration of hydroxyl (OH) is responsible for the band at 3,500–3,400 cm^−1^ (Verma et al., 2014; Raveesha et al., 2019; Amina et al., 2020). In the FTIR spectrum of C-dots the O–H/N–H group is responsible for the absorption peak at 3,407 cm^−1^, while the sharp peak located at 2,925 cm^−1^ is associated with the methyl or methylene (C–H) groups. The characteristic absorption peaks of the CO and COO–functional groups of C-dots are located at 1,593 and 1,403 cm^−1^, respectively. The C–N stretching is responsible for the peaks at 1,260–1,240 cm^−1^, while the C–O/SO stretching vibration is responsible for the peak at 1,033 cm^−1^. Hydroxyl groups (O–H) are present in the C-dots, which contributes significantly to the antibacterial activity of these materials (Chaudhary et al., 2020; Pandiyan et al., 2020; Wang et al., 2020). The FTIR spectra of pure MgO and the C-dots–NPs were compared, and the results showed that the C-dots were conjugated to the surface of the MgO NPs. The band at 1,574 cm^−1^ is related to the bending mode of the NH- vibration MgO–C-dots–PEG. The band at 1,415 cm^−1^ corresponds to the OH deformation band of the phenyl skeleton. The band corresponding to the C–O–C ether stretching vibration absorption is at 1,110 cm^−1^, and the band at 2,885 cm^−1^ corresponds to the C–H stretching vibration (Alas and Genc, 2017; Lou et al., 2020). These results confirm that PEG is covalently bonded to MgO-C-dots NPs. In the FTIR spectrum of pure DOX, the N-H stretching vibration is evident at 3,450 cm^−1^, whereas the O-H stretching vibration is at 3,330 cm^−1^. Both the C-H and C-O stretching vibrations contribute to the maxima at 2,932 and 1730 cm^−1^. The absorption band for N-H bending vibration is located at 1,618 cm^−1^, whereas the band for C-O-C stretching vibration is located at 1,280 cm^−1^ (Cheng et al., 2013; Tian et al., 2017). The FTIR spectra of MgO-C-dots-PEG-DOX showed several of these peaks, confirming DOX attachment to MgO-C-dots-PEG. Overall, the FTIR spectra confirmed that the nano-carrier system included all of the expected component parts.

### 3.4 Spectroscopy

**FIGURE 8:**
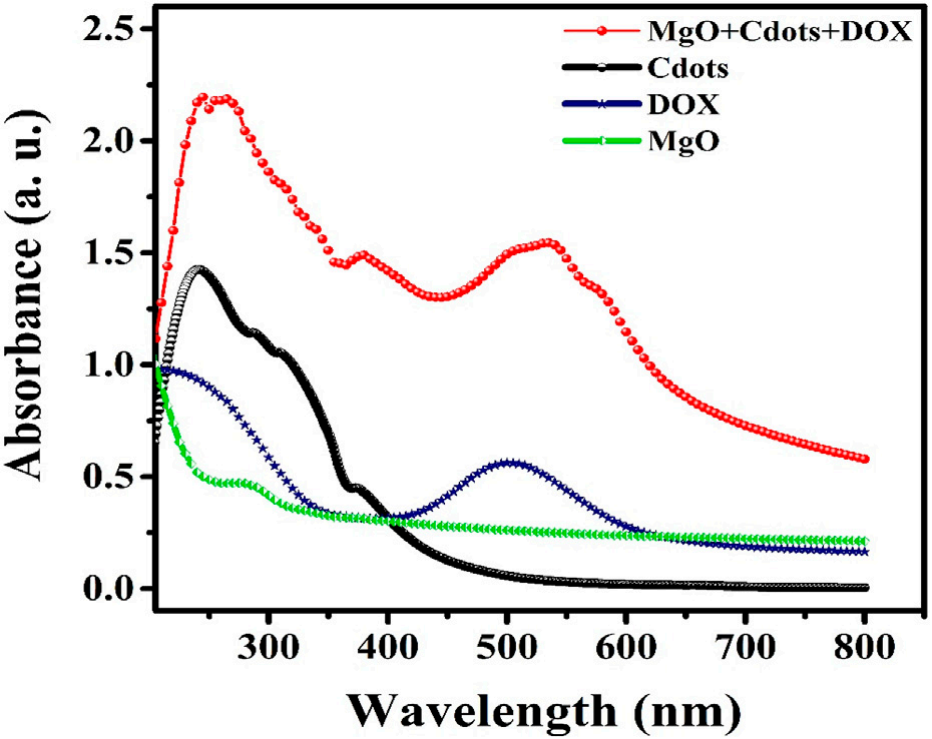
UV/vis spectra of MgO, DOX, C-dots, and MgO/C-dots/DOX.

We recorded the UV-visible spectra of MgO, DOX, C-dots, and MgO/C-dots/DOX NPs from 200 to 800 nm using a UV-vis spectrophotometer to determine their absorbance. The spectra showed that *A. absinthium* L contributed to the production of MgO NPs due to a peak at 285 nm Figure 8 (Almontasser et al., 2019; Alarfaj and El-Tohamy, 2020; Dabhane et al., 2022). The loading of DOX onto MgO/C-dots NPs was confirmed by the UV-vis spectra. The pure MgO, DOX, and C-dots samples all exhibited the same characteristic absorption at 285 nm, 495 nm, and 382 nm, as shown in Figure 8 (Mehn et al., 2017; Pandiyan et al., 2020). MgO/C-dots/DOX showed an absorption maximum around 500 nm (Figure 8). Comparing the spectrum to the spectrum of free DOX, the spectrum had a small shift to a higher wavelength. This is related to the different ways that aggregated doxorubicin molecules absorb light.

### 3.5 FRET

Many fluorescent probes show a relatively small Stokes shift (less than 30 nm), which causes them to self-quench since the molecules absorb their own emitted light. The difficulty can be solved by using a technique known as fluorescence energy transfer (FRET), which produces a much larger pseudo-Stokes shift via the use of donor-acceptor energy transfer (Ren et al., 2018). The term “FRET” refers to the physical process that involves the transfer of energy from one excited molecular fluorophore (donor) to another excited molecular fluorophore (acceptor) in a non-radiative manner. Both long-range dipole–dipole interactions between the molecules, as well as through a non-conjugated spacer could be viable explanations for how this may take place. It is necessary for the emission produced by the donor to overlap with the light absorbed by the acceptor, and it is also necessary for the acceptor and donor to be sufficiently close to each other (about 1–10 nm) (Yuan et al., 2013).

**FIGURE 9:**
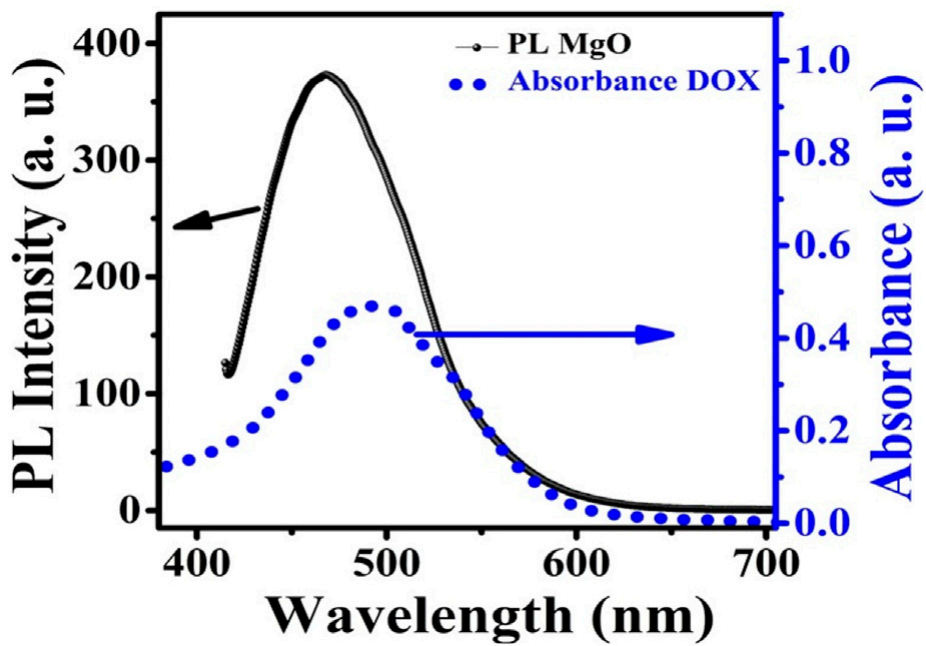
PL emission spectra of MgO NPs superimposed on DOX absorption.

In the present case, it is proposed that when the MgO NPs are excited by 250 nm UV light they show a wide emission band extending from 380 to 600 nm peaking at 480 nm. The absorption band of DOX is centered at 498 nm, which overlaps with the emission band of MgO NPs (Figure 9). As a consequence, energy may be transferred from MgO NPs to DOX molecules in an efficient FRET process. This energy transfer may result in the production of singlet oxygen from the triplet state of DOX.

**FIGURE 10:**
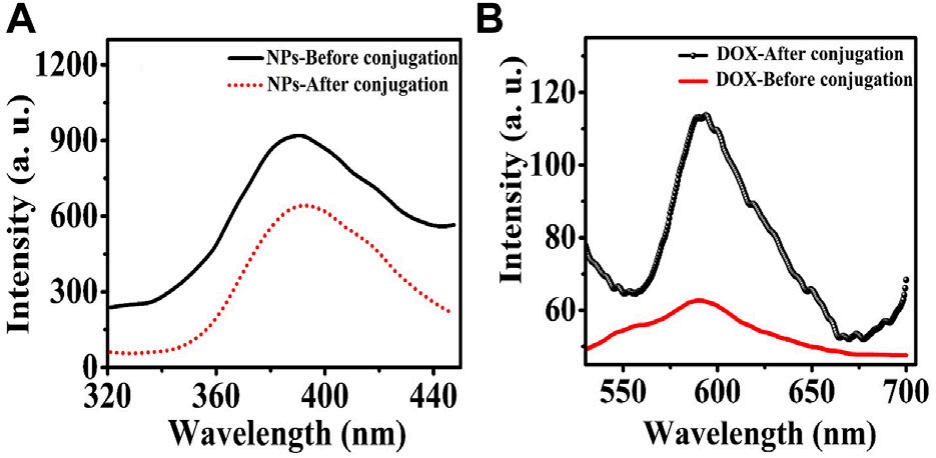
Fluorescence emission spectra of **(A)** MgO NPs and **(B)** DOX before and after binding to each other after excitation at 250 nm wavelength.

The use of FRET showed that MgO NPs (donor) were successfully conjugated to the photosensitizer DOX (acceptor). In Figures 10A, B, the fluorescence emission spectra of the acceptor and donor are shown both pre- and post-conjugation. The excitation wavelength was 250 nm, and emission spectra were obtained over 320–440 nm and 550–710 nm, respectively, for both the donor and the acceptor. In comparison with MgO NPs, the fluorescence of the MgO/C-dots/DOX nanocomposite was reduced by about 20% in Figure 10A. This suggests that energy was transferred from the NPs to the DOX photosensitizer. When compared to the emission from DOX, the emission of MgO/Cdot/DOX nanocomposites, peaking at around 590 nm, increased by approximately 80%, which supports the conjugation of DOX to MgO NPs (Figure 10B).

### 3.6 Phosphorescence mechanism

When discussing the efficacy of PDT, the light penetration depth is often cited as a crucial determining factor. In PDT, the light penetration depth at the appropriate wavelength can be restricted to just a few millimeters at short wavelengths, therefore this PDT can only be used on the surface of the skin, despite the many benefits it offers (Kalka et al., 2000; Fan et al., 2021).

**FIGURE 11:**
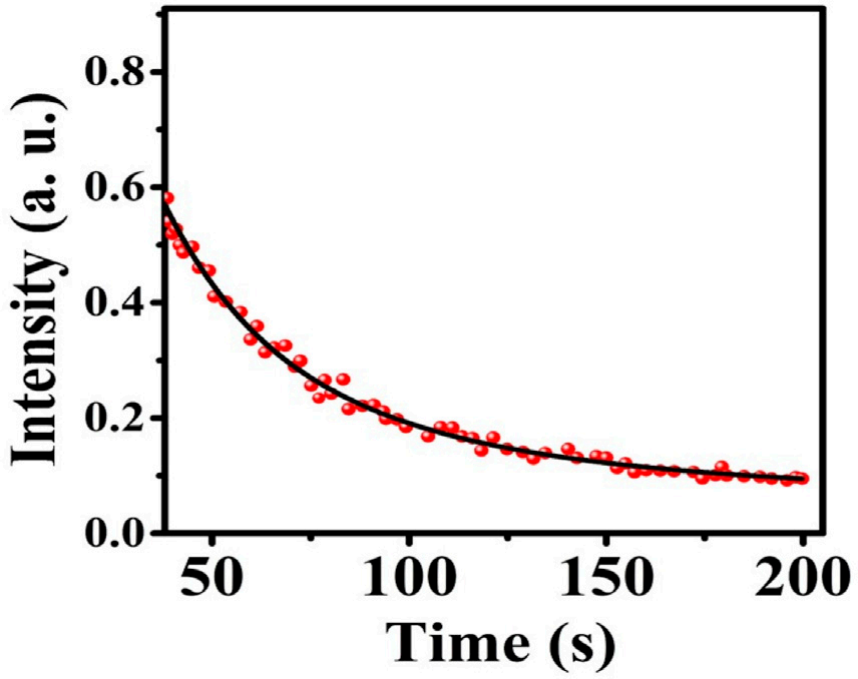
MgO NPs afterglow.

Figure 11 shows the afterglow plot of the MgO NPs. Following activation for 10 min by UVA light in order to transfer excitons into shallow traps from deep ones, the sample was examined for phosphorescence decay at 30°C at 200 s intervals. Figure 11 shows a persistent afterglow. The light intensity rapidly decreased over about 20 s before remaining almost constant for 200 s. As a consequence, it could be possible to excite the MgO/C-dots/DOX nanocomposite with UVA light from outside the body and then rapidly inject them into a tumor for *in vivo* PDT.

### 3.7 Photochemical mechanisms

PDT can operate via two different mechanisms, either type II or type I. The type II mechanism involves singlet oxygen produced by energy transfer from the long-lived triplet state of the photosensitizer to ground-state molecular oxygen. The type I mechanism involves an electron transfer from the excited photosensitizer (singlet or triplet) to molecular oxygen to produce superoxide anion, hydroxyl radicals, and other free radicals. This type I mechanism is broadly similar to the mechanism of photocatalysis, which also involves an electron transfer to oxygen, this time from the high-energy band of semiconductor nanoparticles. Because these reactive oxygen species (ROS) have such a short lifetime, they are difficult to identify. Owing to high sensitivity and ease of data gathering, the use of fluorescence probes is an effective tool for measuring different ROS (Tanaka et al., 2001; Itoo et al., 2022).

To determine the singlet oxygen generation and indirectly assess the cytotoxicity created by the light-activated NPs, anthracene (C14H10) was utilized. Anthracene is converted into anthraquinone by singlet oxygen oxidation, in either the liquid or gas phase. On the other hand, methylene blue (C16H18N3SCl) can be used for detecting hydroxyl radicals and indirectly assessing the cytotoxicity caused by the NPs. The phenothiazinium dye known as methylene blue is very soluble in water, and it is able to preserve its optical properties even after being irradiated with ultraviolet light. After its degradation during the radical oxidation reaction, it is ultimately converted into carbon dioxide and water (Zahedifar et al., 2016). When the color of methylene blue is changed by oxidation, both the emission and the absorption of light are reduced and can be measured (Sadeghi et al., 2019; Roeinfard et al., 2020).

**FIGURE 12:**
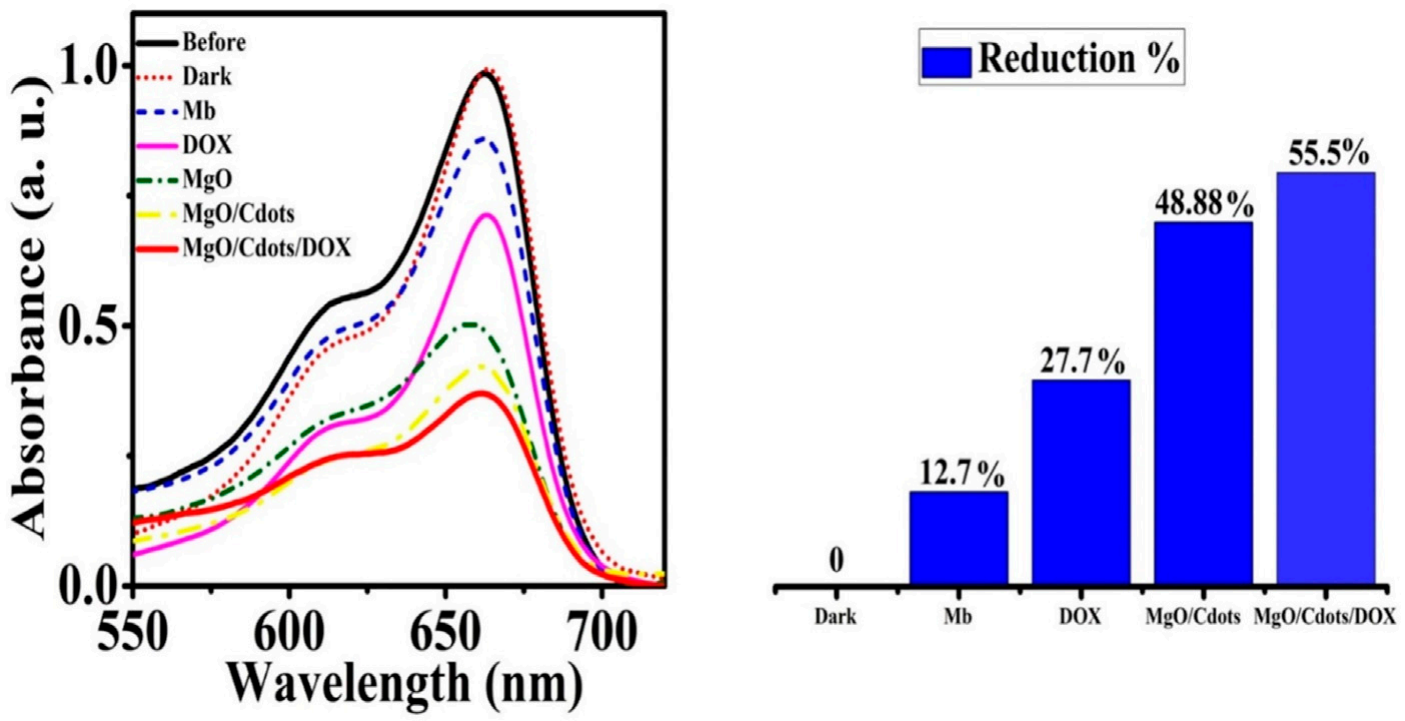
Reduction of MB absorbance after 30 min of UVA irradiation in the presence of DOX, MgO NPs, MgO/C-dots, and MgO/C-dots/DOX.

The generation of hydroxyl radicals in methylene blue solution in the absence and presence of DOX, MgO, MgO/C-dots, and MgO/C-dots/DOX NPs was evaluated after UV excitation. The results are shown in Figure 12. As predicted, irradiating the methylene blue solution in the absence of NPs for 30 min had little effect on its absorption. However, the addition of NPs remarkably decreased the absorption, showing that free radical generation led to the breakdown of methylene blue dye. Figure 12 shows the percentage decrease of methylene blue absorption after irradiation in the presence of control, DOX, MgO/C-dots, and MgO/C-dots/DOX NPs leading to 12.7, 27.7, 48.88, and 55.5%, respectively.

**SCHEME 3:**
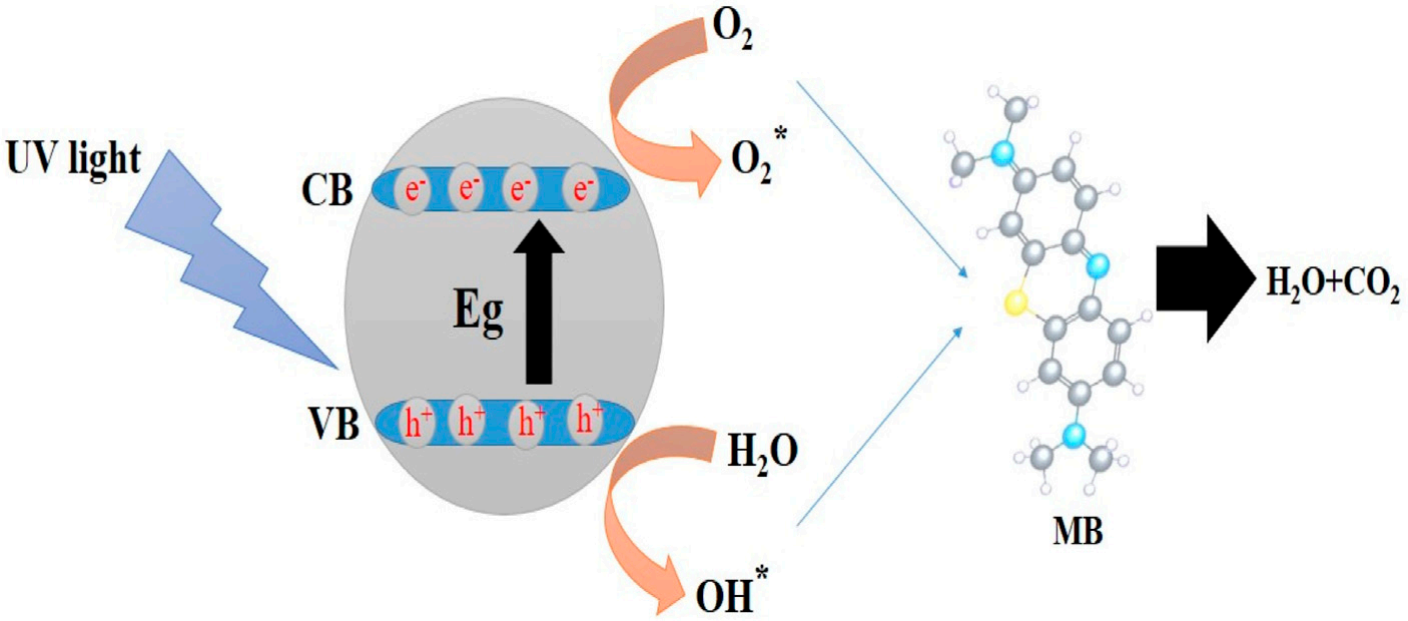
Mechanism for the photocatalytic degradation of MB dye using NPs.

Scheme 3 shows the photocatalysis reaction that causes MB dye to discolor. Electron-hole (e^−^/h^+^) pairs are produced when photons of the proper wavelength are used to illuminate the MgO NPs (Kumar et al., 2022). As soon as the photocatalyst is exposed to UV light, electrons (e^−^) are excited from the valence band to the conduction band and react with oxygen at the surface to form superoxide ions (O_2_^−^), which are then protonated to produce HOO^●^ radicals. Parallel to this oxidation process, the valence band holes (h+) interact with hydroxide anions to create hydroxyl radicals (Banerjee et al., 2014). The reaction may be summed up as follows:

$$\text{MgO}+h\nu \to \text{MgO}\left(h_{\text{vb}}^{+}+e_{\text{cb}}^{-}\right)$$ (1b)

$$\text{MgO}\left(h_{\text{vb}}^{+}+e_{\text{cb}}^{-}\right)\to \text{MgO}+\text{heat}$$ (2)

$$\text{MgO}+H_{2}O_{\text{ads}}\to \text{MgO}+H^{+}+OH^{-}$$ (3)

$$e_{\text{cb}}^{-}+O_{\text{ads}}\to O_{2}^{-•}$$ (4)

$$h_{\text{vb}}^{+}+OH^{-}\to HO_{\text{ads}}^{•}\to HO^{•}+\text{Organic}\to H_{2}O,CO_{2}$$ (5)

**SCHEME 4:**
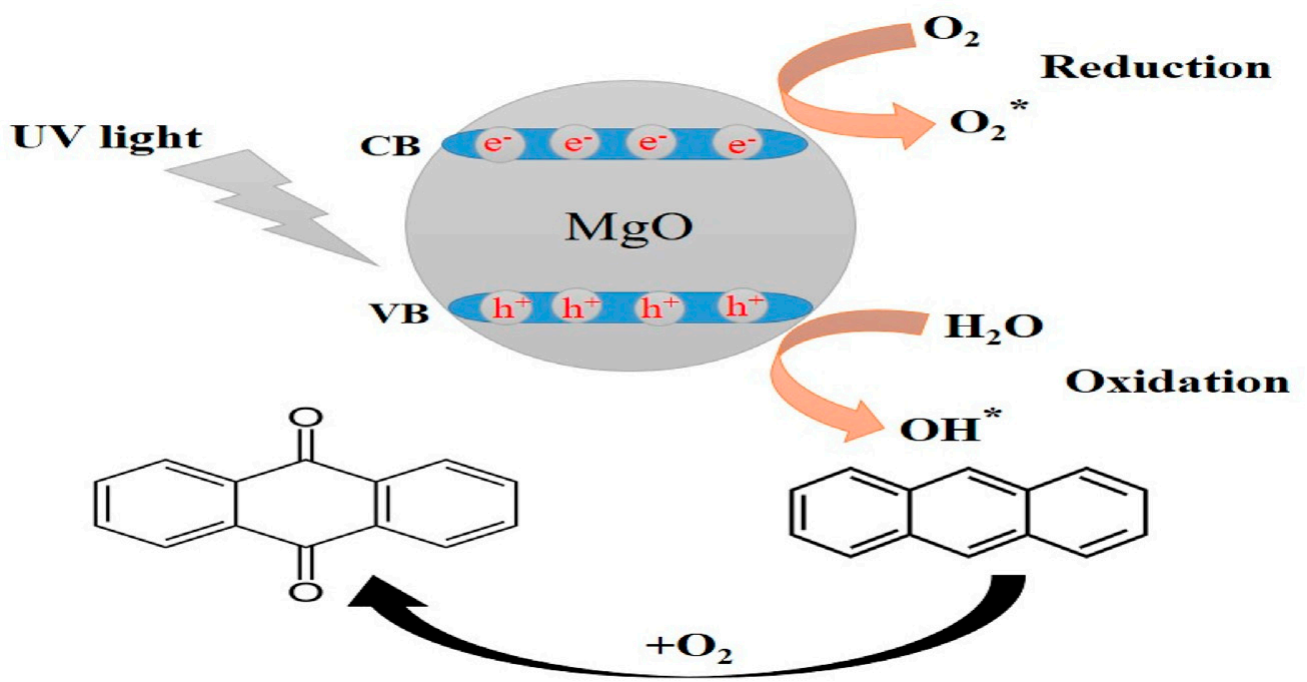
The whole point of using anthracene is that it is somewhat specific for singlet oxygen that first produces an endoperoxide and then anthraquinone.

**FIGURE 13:**
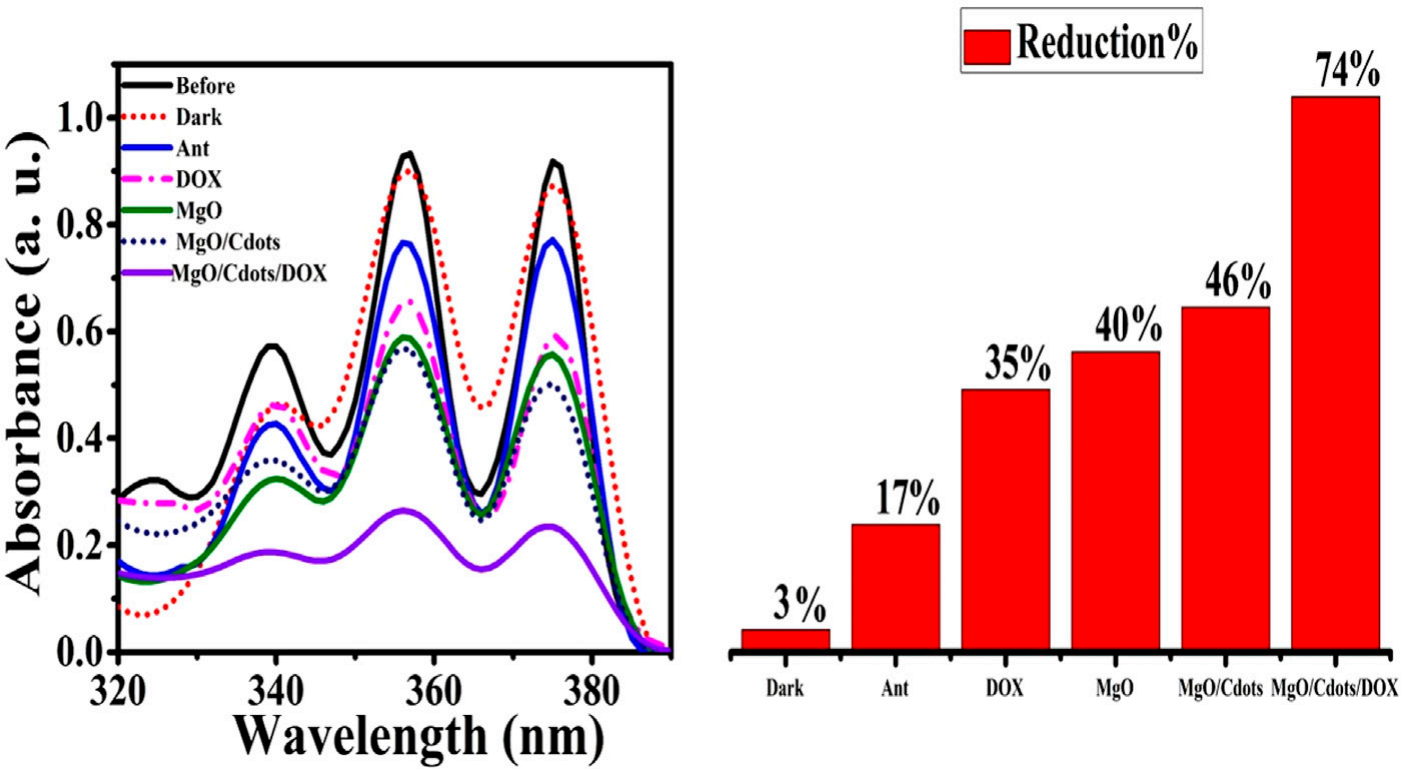
Reduction in intensity of anthracene absorbance by UVA light irradiation for 30 min in presence of free DOX, MgO NPs, MgO/C-dots, MgO/C-dots/DOX.

Figure 13 shows the UV-Vis spectra for a solution of anthracene irradiated in the absence of NPs, as well as in the presence of DOX, MgO, MgO/C-dots, and MgO/C-dots/DOX. There was no significant difference pre and post-irradiation in pure anthracene because it is unable to create singlet oxygen. The anthracene solution irradiated in the presence of NPs showed reduced absorbance owing to anthracene conversion into anthraquinone. The percentage reduction of anthracene for DOX, MgO, MgO/Cdots, and MgO/Cdots/DOX NPs was 35, 40, 46, and 74%, respectively. A schematic of the whole point of using anthracene is that it is somewhat specific for singlet oxygen that first produces an endoperoxide and then anthraquinone can be seen in Scheme 4.

### 3.8 *In vitro* light-mediated cell killing

**FIGURE 14:**
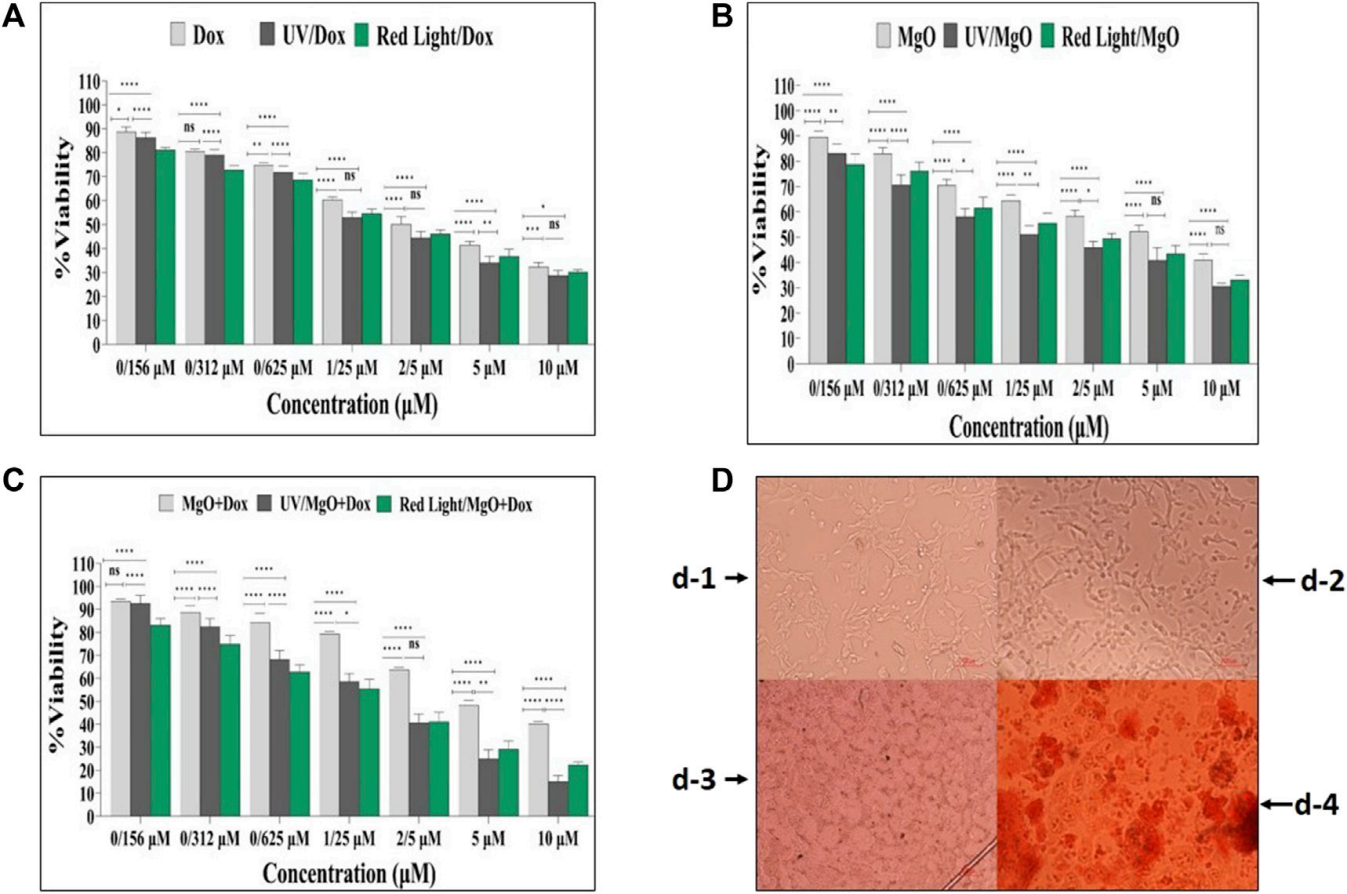
Cell viability of C26 cells. **(A)** Free DOX, DOX + UV light, DOX + Red light. **(B)** MgO, MgO + UV light, MgO + Red light. **(C)** MgO/C-dots/DOX, MgO/C-dots/DOX + UV light, MgO/C-dots/DOX + Red light. **(D)** Microscopic images of (C26) cells before and after treatment with nanoparticles, drugs and irradiation. For comparing more than two groups, ANOVA was used. The statistical significance was set at *p < .*05.

To assess the effects of DOX, MgO NPs, and MgO/C-dots/DOX excited by UV or red light on the viability of C26 cells, we first treated C26 cells with a series of concentrations of each type of PS for 24 h and then irradiated with either UV or red light, followed by measurement of cell viability. As shown in Figure 14A, DOX, DOX + UV, and DOX + Red all significantly reduced the viability with increasing dosage (*p* < 0.0001). DOX + UV were somewhat more effective in killing the cells compared to DOX and DOX + Red. Figure 14B shows that MgO, MgO + UV, and MgO + Red significantly reduced the viability with increasing dosage (*p* < 0.0001). Figure 14C shows that MgO/C-dots/DOX, MgO/C-dots/DOX + UV, and MgO/C-dots/DOX + Red significantly decreased the viability with increasing dosage (*p* < 0.0001). The resulting microscopic images also show a significant change in the shape of the cells. In the Figure 14D-1 there are control cells that are all alive. In Figure 14D-2 after adding NPs, the percentage of survival has decreased from the previous stage, and in Figure 14D-3 the percentage of survival has decreased significantly with the addition of NPs and drugs. And in the Figure 14D-4 more than 70% of cancer cells have been destroyed by irradiating light. With the use of NPs and drugs and irradiation with red and UV light sources, the shape of the cells has changed more than the previous states and has gone out of the normal state, and the number and density of the cells have also decreased. Due to the rounding of the cells, there is a possibility that the cells have undergone apoptosis.

**FIGURE 15:**
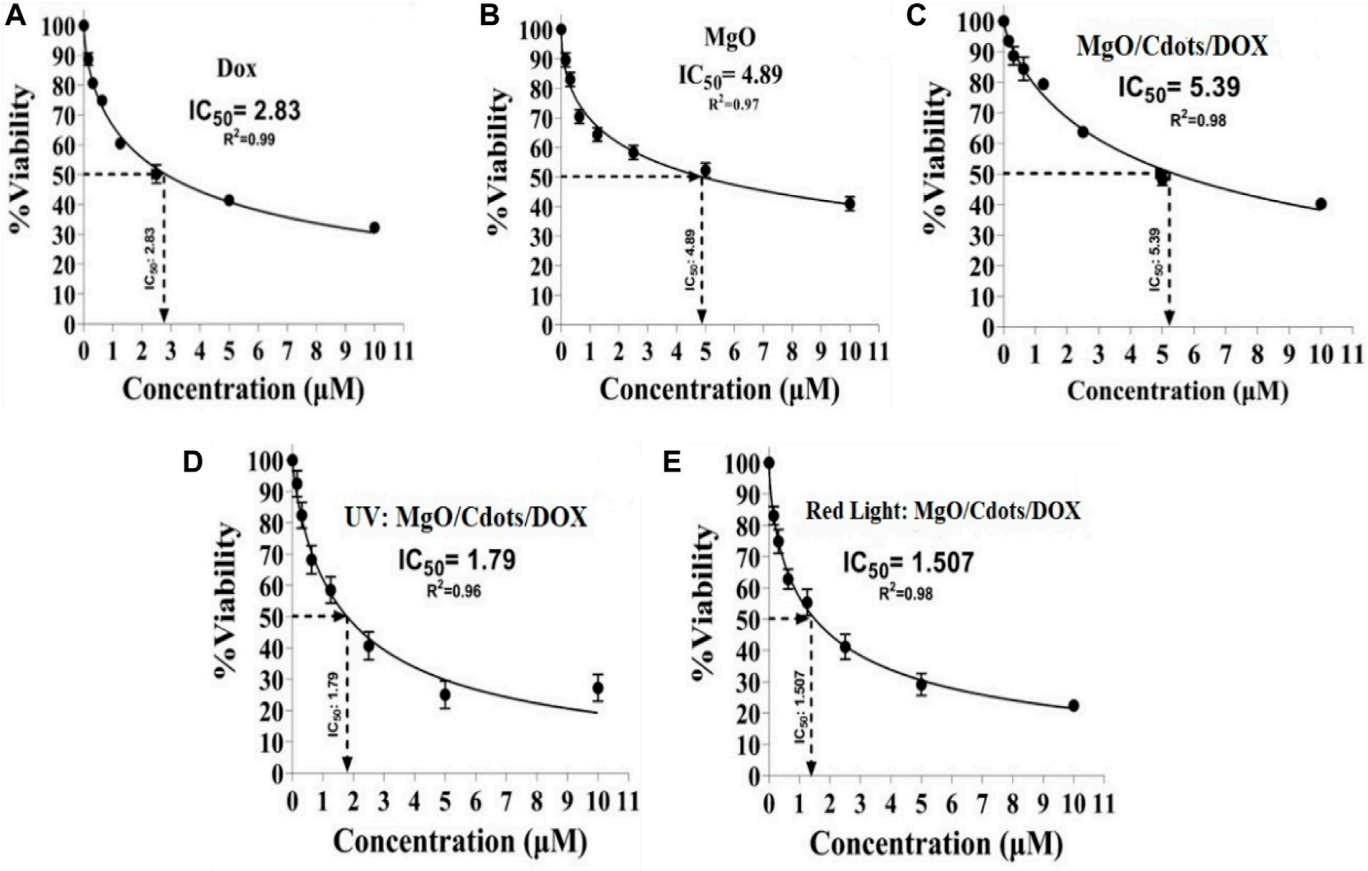
IC50 values of different groups. C26 cells were treated with **(A)** DOX, **(B)** MgO, **(C)** MgO/C-dots/DOX. Cells treated with MgO/C-dots/DOX and irradiated with **(D)** UV light or **(E)** Red light.

Figure 15 shows the calculation of the IC50 values for the various treatment groups on C26 cells. The IC50 concentration for DOX was 2.83 µM (Figure 15A), for MgO it was 4.89 µM (Figure 15B), for MgO/C-dots/DOX it was 5.39 µM (Figure 15C), for MgO/C-dots/DOX + UV light it was 1.79 µM (Figure 15D), and for MgO/C-dots/DOX + Red light it was 1.507 µM (Figure 15E).

## 4 Conclusion

In this work, MgO NPs and MgO/C-dots nanocomposites were prepared by green synthesis and characterized. The anticancer drug DOX was loaded onto the nanocomposites. MgO and MgO/C-dots showed colloidal stability and good biocompatibility. There was evidence of a FRET mechanism taking place by which UV irradiation could transfer energy to DOX. This FRET process allowed the generation of type I photocatalysis ROS from the MgO NPs as well as type 2 singlet oxygen from the DOX photosensitizer under UV irradiation. In the *in vitro* PDT experiments red light irradiation could be employed to produce phototoxicity to the C26 cells by absorption by the DOX molecules. These nanocomposites could be used to carry out PDT of superficial cancers such as gastro-intestinal tumors and skin cancer. Our budget was limit. In this regard, we suggest to use HPLC for analyzing the profile of the used plant. And use the XPS for quantitatively analysis in future studies.

## Data Availability

The original contributions presented in the study are included in the article/supplementary material, further inquiries can be directed to the corresponding author.
